# Supplementary material for: Bat detective—Deep learning tools for bat acoustic signal detection
Source: PLoS Comput Biol. 2018 Mar 8;14(3):e1005995. doi: 10.1371/journal.pcbi.1005995 (PMC5843167; doi:10.1371/journal.pcbi.1005995)
Supplement: S1 Text — Description of the CNN architectures, training details, and information about how the training data was collected. (PDF) [file pcbi.1005995.s001.pdf]

S1 Text

CONVOLUTIONAL NEURAL NETWORK DETECTORS

The full Convolutional Neural Network (CNN_{FULL}) was made up of three convolution layers, each consisting of 32 3x3 filters, rectified linear nonlinearities (ReLU) [1], and 2x2 max pooling. The last pooling layer was fed into a fully connected layer with 256 units, which was followed by an output of two softmax units. Both the fully connected layer and softmax layer utilised dropout [2] during training. This resulted in over 25,000 weights that were used to learn from the data. The network was trained for 50 epochs, with a batch size of 256 using Nesterov’s variant of stochastic gradient descent with a fixed learning rate and momentum of 0.01 and 0.9, respectively. Our training data only contained annotations for the points in time where bat search-phase calls occurred. To artificially increase the training set, we selected additional positive training examples 1.5 ms either side of each positive annotation. To generate an initial negative training set, we sampled random temporal windows that were not labelled as containing search-phase echolocation calls. We sampled the same number of negatives as positives. During training, we performed two rounds of hard negative mining to add more challenging negative examples to the training set [3]. This resulted in a combined training set of 32,000 temporal windows. A time window was considered a hard negative if it did not occur within 7ms of an annotated search-phase bat call and was incorrectly predicted as containing a search-phase call with a probability of greater than 0.5. We found this step to greatly reduce the number of false positive detections as it gave the CNN access to temporal windows that it initially incorrectly classified.

CNN_{FAST} increased the test time evaluation speed of CNN_{FULL} in two main ways. First, it had fewer parameters, with two rather than three convolution layers, each made up of 16 3x3 filters (Supplementary Information Figure 1). Again, both convolution layers were followed by ReLUs and 2x2 max pooling, and the fully connected layer consisted of only 64 units. The second performance improvement exploited the fact that the convolutional layers could be run on the entire spectrogram without having to extract out individual time windows [4, 5]. To do this, we converted the fully connect units to convolution units and applied them across the whole spectrogram. CNN_{FAST} was trained in the same fashion as CNN_{FULL}, as only the test time performance needed to be efficient.

Our system was implemented in Python 2.7 [6], heavily utilising vectorized instructions from Numpy [7]. Portions of the code that were inefficient in pure Python (e.g. non-maximum suppression) were implemented in Cython [8]. To train the CNNs, we used the Lasagne library [9] which is a wrapper around Theano [10]. To further improve training efficiency, we made use of the Cuda [11] and cuDNN [12] libraries. Training the large CNN took on the order of 1.5 hours on a desktop machine with an Intel i7 processor, 32Gb of RAM, and a Nvidia GTX 1080 GPU.

INDICATOR BATS PROGRAMME PROTOCOLS

Citizen scientists within the iBats programme [13] collected 2501 full-spectrum acoustic approximately 90 minute recordings along 40km road transects routes driven at 25 km/h and starting 30-45 minutes after sunset, twice yearly during July to August (the period of peak seasonal activity for most northern European species) spanning a period from 2005-2015 across 14 countries (UK, Romania, Bulgaria, Hungary, Ukraine, Russia,

Mongolia, Japan, Thailand, Ghana, Zambia, New Zealand, Australia, and USA). Acoustic data was recorded with a time-expansion (TE) device equipped with a wideband capacitance microphone with the frequency range 10-160 kHz and a sample rate of 409.6 kHz (Tranquility Transect, Courtplan Design Ltd, UK). The recording devices were set to record using a TE factor of 10, sampling time of 320 ms, sensitivity set on maximum, giving a continuous sequence of ‘snapshots’, consisting of 320 ms of silence (sensor listening) and 3.2s of TE audio played backed 10 times slower than real time. The recording device was attached to the front or back passenger window of the vehicle with the microphone pointing up and back at a 45° angle. Sony MiniDiscs (Sony-MZ-NH600 Silver-Hi-MiniDisc Walkman), Zoom H2s, Edirol R-09HR or Roland R-05 recording devices were used to record audio without compression or using a lossless compression, and native files were transformed into a wav format. A GPS track of the transect route was recorded using a variety of different GPS options, for example PDAs (Mio A201, Mio 350, Mio 360) with a built in Global Positioning System (GPS) taking a reading every 5 seconds via a mapping software program (GPSTuner v.4 and v.5), or a basic GPS (Garmin Etrex Vista, Garmin Etrex Legend HCx), and the transect tracks were recorded as GPX files. The sound recorder and GPS track were set to start recording at the same time so that the position of the car when each bat is recorded could be determined. Each recorded bat call was subsequently georeferenced using this GPS track. Surveys were only carried out during ‘fine’ weather; i.e. when the air temperature was greater than 7°C, and no more than very light rain or wind. The following metadata were also recorded at the start and end of each transect:

temperature (°C); cloud cover (%); rain (dry, drizzle, light); and wind speed (calm, light, or breezy).

References

1. Nair V, Hinton GE, editors. Rectified linear units improve restricted boltzmann machines. Proceedings of the 27th International Conference on Machine Learning (ICML-10); 2010.
2. Srivastava N, Hinton GE, Krizhevsky A, Sutskever I, Salakhutdinov R. Dropout: a simple way to prevent neural networks from overfitting. Journal of Machine Learning Research. 2014;15(1):1929-58.
3. Sung K-K, Poggio T. Example-based learning for view-based human face detection. IEEE Transactions on pattern analysis and machine intelligence. 1998;20(1):39-51.
4. He K, Zhang X, Ren S, Sun J, editors. Spatial pyramid pooling in deep convolutional networks for visual recognition. European Conference on Computer Vision; 2014: Springer.
5. Girshick R, editor Fast r-cnn. Proceedings of the IEEE International Conference on Computer Vision; 2015.
6. Python Software Foundation. Python 2.7 Programming Language.
7. Van Der Walt S, Colbert SC, Varoquaux G. The NumPy array: a structure for efficient numerical computation. Computing in Science & Engineering. 2011;13(2):22-30.
8. Behnel S, Bradshaw R, Citro C, Dalcin L, Seljebotn DS, Smith K. Cython: The best of both worlds. Computing in Science & Engineering. 2011;13(2):31-9.
9. Dieleman S, Schlüter J, Raffel C, Olson E, Sønderby SK, Nouri D, et al. Lasagne: First Release. 2015.
10. Bergstra J, Breuleux O, Bastien F, Lamblin P, Pascanu R, Desjardins G, et al., editors. Theano: A CPU and GPU math compiler in Python. Proc 9th Python in Science Conf; 2010.
11. Nickolls J, Buck I, Garland M, Skadron K. Scalable parallel programming with CUDA. Queue. 2008;6(2):40-53.
12. Chetlur S, Woolley C, Vandermersch P, Cohen J, Tran J, Catanzaro B, et al. cudnn: Efficient primitives for deep learning. arXiv preprint arXiv:14100759. 2014.
13. Jones KE, Russ JA, Bashta A-T, Bilhari Z, Catto C, Csősz I, et al. Indicator Bats Program: A System for the Global Acoustic Monitoring of Bats. Biodiversity Monitoring and Conservation: Wiley-Blackwell; 2013. p. 211-47.
14. Szewczak JM. Sonobat 2010.
15. Bates D, Mächler M, Bolker B, Walker S. Fitting Linear Mixed-Effects Models Using lme4. 2015. 2015;67(1):48. Epub 2015-10-07. doi: 10.18637/jss.v067.i01.
