# Supplementary figures and images for: Bat detective—Deep learning tools for bat acoustic signal detection
Source: PLoS Comput Biol. 2018 Mar 8;14(3):e1005995. doi: 10.1371/journal.pcbi.1005995 (PMC5843167; doi:10.1371/journal.pcbi.1005995)

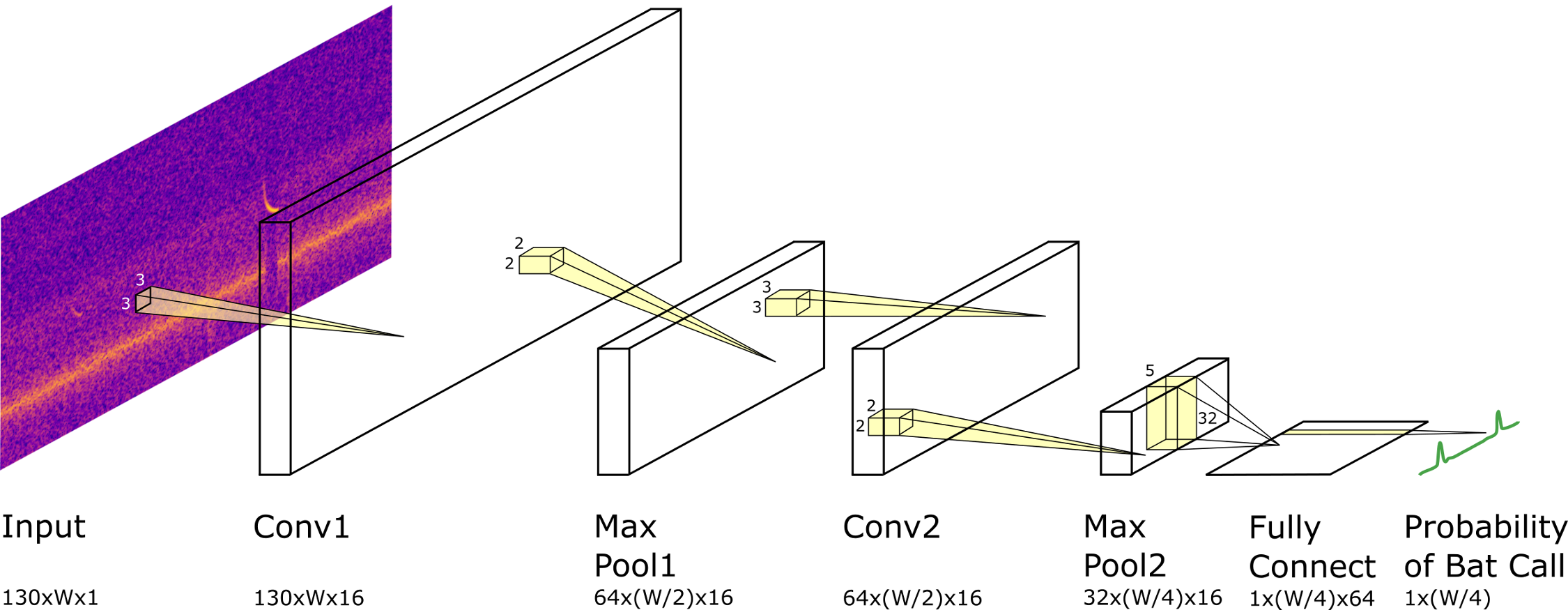

Supplement: S1 Fig — The CNNFAST network consists of two convolution layers (Conv1 and Conv2), with 16 filters each (shown in yellow, with the filter size shown inset). Both convolution layers are followed by a max pooling layer (Max Pool1 and Max Pool2), and the network ends with a fully connected layer with 64 units (Fully Connect). CNNFAST computes feature maps (shown as white boxes) across the entire input spectrogram, resulting in less computation and a much faster run time. The fully connected layer is also evaluated as a convolution. The output of the detector is a probability vector (shown in green) whose length is one quarter times the width of the input spectrogram. The numbers below each layer indicate the height, weight, and depth of the corresponding layer. (TIF) [file pcbi.1005995.s002.tif]

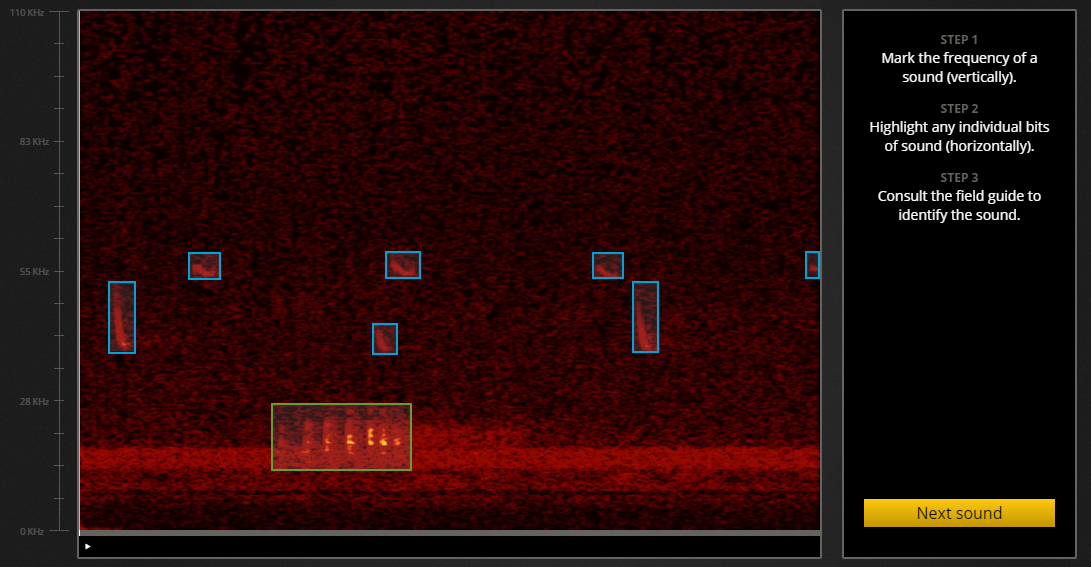

Supplement: S2 Fig — Boxes represent example user annotations of sounds in a spectrogram of a 3840ms sound clip, showing annotations of two sequences of search-phase echolocation bat calls (blue boxes), and an annotation of an insect call (yellow box). (TIF) [file pcbi.1005995.s003.tif]

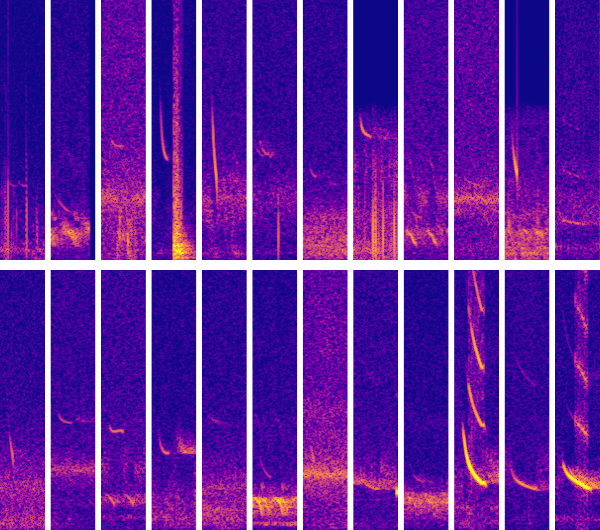

Supplement: S3 Fig — Each example is represented as a spectrogram of duration 23 milliseconds and frequency range from 5–115 kHz using the same FFT parameters as the main paper, and contains examples of different search-phase echolocation call type, but also a wide variety of background non-bat biotic, abiotic and anthropogenic sounds. (TIF) [file pcbi.1005995.s004.tif]
