# Supplementary material for: Bat detective—Deep learning tools for bat acoustic signal detection
Source: PLoS Comput Biol. 2018 Mar 8;14(3):e1005995. doi: 10.1371/journal.pcbi.1005995 (PMC5843167; doi:10.1371/journal.pcbi.1005995)
Supplement: S1 Table — TE represents time-expansion recordings (x10); RT real-time recordings. Note that the length of the clips is approximately comparable for both the iBats and the Norfolk Bat Survey data as the total iBats clip length of 3.84s corresponds to 320ms of ultrasonic sound slowed down ten times (3.2s) and buffered by silence on either side. (PDF) [file pcbi.1005995.s005.pdf]

S1 Table

Test Dataset	iBats (Romania & Bulgaria)	iBats (UK)	Norfolk Bat Survey (Norfolk, UK)
Recording device	Tranquility Transect	Tranquility Transect	SM2 Song Meter
Recording type	TE, full spectrum	TE, full spectrum	RT, full spectrum
Recording method	Road transect	Road transect	Stationary Point
No. of clips	500	434	500
Mean duration	3.84s	3.84s	400ms
No. of bat calls	1604	842	1345
