# Supplementary material for: Bat detective—Deep learning tools for bat acoustic signal detection
Source: PLoS Comput Biol. 2018 Mar 8;14(3):e1005995. doi: 10.1371/journal.pcbi.1005995 (PMC5843167; doi:10.1371/journal.pcbi.1005995)
Supplement: S2 Table — β represents slope, Std standard deviation, Z Z-value, p probability. Analytical systems compared were SonoBat (version 3.1.7p) [14] and BatDetect CNNFAST, using a 0.9 probability threshold. Data from using acoustic monitoring data collected from Jersey, UK between 2011–2015. See main text for definition of a bat pass. GLMMs were fitted using lme4 [15] with model formula: detections ~ analytical_method + (1|sampling_event) + (1|transect) + (1|date). (PDF) [file pcbi.1005995.s006.pdf]

S2 Table

	Call Detections			Pass Detections		
Fixed Effects	**β (Std)**	**Z**	**p**	**β (Std)**	**Z**	**p**
Intercept	4.31 (0.09)	46.6	$<2e^{-16}$	3.28 (0.08)	41.9	$<2e^{-16}$
Analytical System	0.37 (0.11)***	34.4	$<2e^{-16}$	0.06 (0.02)	3.2	0.00143
Random Effects	**Variance (Std)**			**Variance (Std)**		
Sampling Event	0.195 (0.44)			0.108 (0.33)		
Transect	0.034 (0.18)			0.035 (0.18)		
Date	0.036 (0.19)			0.018 (0.13)		
